# Using the drug repositioning approach to develop a novel therapy, tipepidine hibenzate sustained-release tablet (TS-141), for children and adolescents with attention-deficit/hyperactivity disorder

**DOI:** 10.1186/s12888-020-02932-2

**Published:** 2020-11-10

**Authors:** Takuya Saito, Yushiro Yamashita, Akemi Tomoda, Takashi Okada, Hideo Umeuchi, Saki Iwamori, Satoru Shinoda, Akiko Mizuno-Yasuhira, Hidetoshi Urano, Izumi Nishino, Kazuhiko Saito

**Affiliations:** 1 Department of Child and Adolescent Psychiatry, Hokkaido University Graduate School of Medicine, Sapporo, Japan; 2 Department of Pediatrics and Child Health, Kurume University School of Medicine, Fukuoka, Japan; 3 Research Center for Child Mental Development, University of Fukui, Fukui, Japan; 4 Department of Developmental Disorders, National Institute of Mental Health, National Center of Neurology and Psychiatry, Tokyo, Japan; 5 Department of Child and Adolescent Psychiatry, Nagoya University Graduate School of Medicine, Nagoya, Japan; 6 Taisho Pharmaceutical Co., Ltd., Tokyo, Japan; 7 Aiiku Counselling Office, Aiiku Research Institute, Imperial Gift Foundation Boshi-Aiiku-Kai, Tokyo, Japan

**Keywords:** Drug repositioning, TS-141, ADHD, CYP2D6 polymorphism, Phenotype, Clinical trial, Tipepidine

## Abstract

**Background:**

Asverin® (tipepidine hibenzate) has been used as an antitussive for > 50 years in Japan. Studies revealed that tipepidine modulates monoamine levels, by inhibiting G-protein-activated inwardly rectifying potassium (GIRK) channels, expecting the potential therapeutic effects of tipepidine for attention-deficit/hyperactivity disorder (ADHD) in recent years. In this study, TS-141, a sustained-release tablet of tipepidine, was developed for the treatment of ADHD through a drug repositioning approach.

**Methods:**

The sustained-release profile of TS-141 in healthy adults was investigated, and tipepidine exposure in the plasma after the TS-141 administration was compared to that of Asverin in the phase I study. Phase II study was conducted to examine the effects of TS-141 30 (once a day), 60 (once a day), 120 mg (60 mg twice a day), or placebo, that is within the exposure in the maximum dosage of Asverin, in children and adolescents with ADHD, and was designed as an 8-week treatment, randomized, parallel group, double-blind, placebo-controlled trial recruiting 6–17-year-old children and adolescents diagnosed with ADHD. A total of 216 patients were randomized according to the CYP2D6 phenotype. The primary end-point was ADHD Rating Scale IV-J changes. Furthermore, effects of CYP2D6 phenotype on the efficacy in the subgroup analysis were investigated.

**Results:**

TS-141 had the sustained-release profile, and the CYP2D6 phenotype had effects on the plasma exposure of tipepidine. ADHD RS-IV-J scores in all TS-141 dosages decreased from their baseline scores; however, no significant difference was observed in ADHD RS-IV-J score changes between the placebo and TS-141-administered groups. In patients with intermediate metabolizer CYP2D6, ADHD RS-IV-J score changes in the 120 mg group tended to be larger than that in the placebo group.

**Conclusions:**

ADHD RS-IV-J changes on TS-141 may depend on the interaction between the TS-141 dose and CYP2D6 phenotype, suggesting that further clinical trials should be conducted with careful consideration of polymorphism. Drug repositioning approach of TS-141 was attempted at the same dose as that of antitussive; however, dose setting according to the indication was necessary.

**Trial registration:**

Phase I study: JapicCTI-205235 (Registered 25 March 2020), Phase II study: JapicCTI-163244 (Registered 9 May 2016), https://www.clinicaltrials.jp/cti-user/trial/Show.jsp

**Supplementary Information:**

**Supplementary information** accompanies this paper at 10.1186/s12888-020-02932-2.

## Background

Drug repositioning/repurposing is defined as the strategy that employed existing compounds with the ultimate aim of successfully registering the drug for a new and potentially patentable indication [1]. Drug repositioning has attracted attention in recent years, and several advantages such as significant reduction of development costs, shortening of development period, and increased success probability were identified [2]. Asverin® (tipepidine hibenzate tablet) is a non-narcotic antitussive agent that has been widely used for > 50 years since its approval in 1959 in Japan and its safety in children and adolescents has already been established. Recently, tipepidine has been shown to attenuate the hyperactivity caused by neonatal 6-hydroxydopamine lesion, which is considered as an animal model of attention-deficit/hyperactivity disorder (ADHD) [3]. Several clinical open pilot studies have recently reported the effects of Asverin tablets for pediatric ADHD [4–6]. Thus, tipepidine is potentially useful as a therapeutic agent for pediatric ADHD. Since the half-life of Asverin is short, approximately 1.8 h in humans (Asverin package insert), leading to three doses a day and may be decreased in adherence for ADHD, we designed the slow release formulation of tipepidine in order to improve adherence to the treatment.

ADHD is characterized by three main symptoms (inattention, hyperactivity, and impulsivity) and is classified as a neurodevelopmental disorder in DSM-5 [7]. In DSM-5, its prevalence has been reported to be 5% in children. According to a nationwide survey for teachers in Japan, the proportion of students who markedly show carelessness or hyperactivity-impulsiveness problems was 3.1% [8]. The quality of life (QOL) of patients with ADHD has been shown to decrease as the ADHD severity increased [9, 10]. Adults with ADHD have psychosocial impairment, poorer occupational performance, substance use, and traffic accidents [7]. Long-term follow-up clinical trials have reported that adult ADHD persistence is estimated approximately 1/3 to 2/3 of patients with ADHD in childhood [11, 12]. Early treatment of ADHD-related symptoms is important to improve the patient’s QOL [13].

Several drugs have already been approved for pediatric ADHD: central stimulants such as methylphenidate hydrochloride and lisdexamfetamine mesilate and non-stimulants such as atomoxetine hydrochloride, guanfacine hydrochloride, and clonidine hydrochloride. However, some concerns on potential long-term effects of stimulant ADHD medications were found on the patients’ growth and weight, and these stimulants have a risk of drug abuse [14]. Conversely, non-stimulants, atomoxetine, have side effects such as nausea, vomiting, and decreased appetite [15]. When using guanfacine, heart rate and blood pressure should be measured prior to the initiation of therapy, followed by dose increase, periodically while on therapy, and occurrence of related symptoms should be considered [16, 17] Because of these problems, a novel ADHD therapeutic agent that can be used more safely is desired [18].

The efficacy of tipepidine for ADHD is expected according to some non-clinical [19] and clinical reports. Although the potential mechanism of tipepidine for ADHD remains unclear, G-protein-coupled inwardly rectifying potassium (GIRK) channel inhibitory action is considered to be one of the mechanisms [20, 21]. Activation of monoamine nerves by GIRK channel inhibition is thought to promote dopamine and noradrenaline release in the nucleus accumbens and frontal cortex [22–24].

Using the drug repositioning development method, tipepidine was tested as a treatment for ADHD within the range of exposures under the doses already approved for antitussive. In this study, the pharmacokinetics of TS-141 was investigated in the phase I study, and its efficacy and safety for pediatric patients with ADHD were evaluated in the phase II study. This report describes what needs to be considered in the drug repositioning development method, for example, the dose setting for new indications and gene polymorphisms of drug-metabolizing enzymes that greatly affect its pharmacokinetics.

## Methods

### Phase I study

#### Study design

This was an open-label, single site, pharmacokinetic study of TS-141 and Asverin in healthy adult men aged 20–39 years in Japan. In this phase I study, TS-141 15, 30, and 60 mg and Asverin 40 mg was administered as a single dose and 60 mg of TS-141 was given twice daily for 7 days. Tipepidine plasma concentrations after administrations of Asverin or TS-141 were determined using liquid chromatography-tandem mass spectrometry methods (LC-MS/MS) methods, and blood samples were analyzed for CYP2D6 genotype by testing the alleles (Luminex xTag CYP2D6v3 RUO, Luminex Japan). Subjects were classified according to 4 phenotypes; ultrarapid metabolizer (UM), extensive metabolizer (EM), intermediate metabolizer (IM), and poor metabolizer (PM). Safety evaluations include adverse events (AEs), side effects, body weight, vital signs, 12-lead electrocardiograms (ECGs), and clinical laboratory parameters.

### Phase II study

#### Study design

**Fig. 1:**
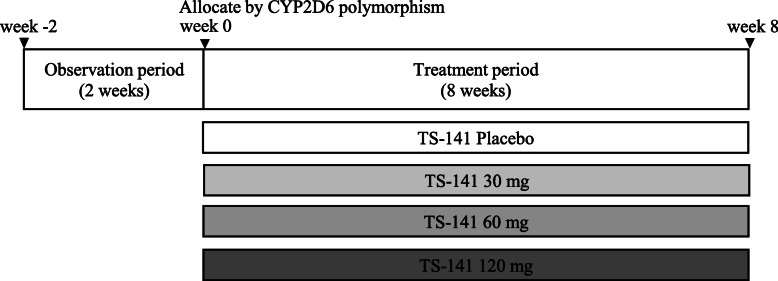
Study Design of Phase II Clinical trial. The study was conducted in 2 periods, a 2-week observation period and an 8-week double-blind treatment period. Participants who meet all study criteria at baseline were randomized in a 1:1:1:1 ratio in a fixed daily dose of 30 mg (once a day), 60 mg (once a day), 120 mg (60 mg twice a day), or placebo. Each patient was randomized for each dose according to CYP2D6 phenotype as a stratification factor: UM and EM group, IM and PM group, or unknown group in CYP2D6 phenotype

This was a randomized, parallel group, double-blind, placebo-controlled phase II study of TS-141 in pediatric patients with ADHD. This trial was conducted at 53 sites in Japan in two periods: a 2-week observation and an 8-week double-blind treatment period (Fig. 1). Participants who meet all study criteria at baseline were randomized by the stratified block randomization method with the CYP2D6 phenotype as the stratification factor in a 1:1:1:1 ratio in a fixed daily dose of 30 mg (once a day), 60 mg (once a day), 120 mg (60 mg twice a day), or placebo. We set the minimum dose at 30 mg, which has been reportedly effective for ADHD in previous open studies, and the maximum dose of 120 mg to utilize the information on the safety of Asverin. Based on the results of the phase I study, each patient was randomized for each dose based on the CYP2D6 phenotype as a stratification factor: UM and EM group, IM and PM group, or unknown group. The trial enrolled outpatients aged 6–17 years. All patients were diagnosed according to the Diagnostic and Statistical Manual of Mental Disorders-5 (DSM-5) criteria for ADHD. Patients who had a total score of ≥23 on ADHD RS-IV-J:I (Investigator) and score of ≥3 on CGI-ADHD-S were included in this study. Then, patients having each of the following items were excluded: history or current diagnosis of schizophrenic disorder or any psychiatric disorder (diagnosed by DSM-5), comorbid of reactive attachment disorder, and intellectual disabilities (defined as intelligence quotient of < 70 by Wechsler scale or Binet scale). DSM-5 allowed the coexistence of autism spectrum disorder (ASD), allowing participation in this trial only when ASD was not dominant (allowed up to level 1 in the DSM-5 diagnostic criteria). To participate in this study, patients were banned from taking any medicine for ADHD medication.

The primary outcome measure was investigator’s evaluation of ADHD Rating Scale IV Japanese version (ADHD RS-IV-J:I) as a valid and widely utilized measurement tool in school-aged children with ADHD [25]. Secondary outcome measures included the Clinical Global Impression-Severity of ADHD (CGI-ADHD-S) and Questionnaire-Children with Difficulties (QCD), a questionnaire completed by parents [26], and ADHD RS-IV-J:School from teachers. An evaluation form was provided to teachers whose cooperation was obtained prior to the study, and then teachers completed the form and mailed back to the study sites before each visit. Investigators comprehensively evaluated the ADHD RS-IV-J:I based on semi-structured interviews with parents and ADHD RS-IV-J:School. The evaluation period was obtained from previous visits to the visit date. All investigators who evaluate the primary end-point were trained, and the reliability of the evaluation was ensured (validated by Bracket Global LLC). In the subgroup analysis, for each CYP2D6 phenotype, changes in primary and secondary end-points were tabulated according to the treatment group. The dose response on ADHD RS-IV-J:I changes were considered for each CYP2D6 phenotype. Safety evaluations include AEs, body weight, vital signs, 12-lead ECGs, Columbia-suicide severity rating scale (C-SSRS), physical examination findings, and clinical laboratory parameters.

### Statistical analysis

In the phase I study, the pharmacokinetic assessment was conducted in patients for whom pharmacokinetic end-points were available without significant protocol deviations such as inclusion and exclusion criteria. The safety evaluation was conducted on patients who had been administered the study drug even once, and the safety end-point was measured even once after the study drug was administered. Summary statistics (e.g., mean, standard deviation) were calculated for Cmax, AUC and MRT as pharmacokinetic parameters. The sample size was determined enough to assess pharmacokinetic profile. Statistical analyses were performed using the software Statistical Analysis System version 9.2.

In the phase II study, statistical analyses were performed using the software Statistical Analysis System version 9.2 and 9.4. All efficacy analyses were performed on patients who met the entry criteria and received the study drug at least once (full analysis set (FAS)) and who had no critical deviation (per protocol set (PPS)). To estimate the sample size, it was assumed that the ADHD RS-IV score changes between placebo and study drug group were 6.0 ± 10.0 (mean ± standard deviation), referring from a past placebo-controlled study in Japanese patients with ADHD [15]. The number of sample size was estimated 200 in the significance level of 0.05 and the power of 0.80.

The primary outcome was changes in ADHD RS-IV-J:I total score from baseline (week 0) until week 8 in the TS-141 groups compared with placebo. If the 8-week data were missing, then they were imputed using the last observation carried forward manner. Changes from baseline among the dose groups were compared using the analysis of covariance (ANCOVA) model with the baseline, dose, and CYP2D6 phenotype as covariates. Subjects whose ADHD-RS-IV:I total score change from baseline decreased by 25% or more were designated as responders, and the response rate was calculated.

## Results

### Phase I study

In 48 subjects, all Japanese who received the study drug, no AEs were observed, and no clinically significant changes such as abnormal laboratory values were observed (range for patient recruitment and follow-up: November 14, 2014 to July 13, 2015). In addition, the plasma concentration of tipepidine was increased in a dose-dependent manner (data not shown) after a single administration of TS-141 15, 30, and 60 mg to the same subject with dose-up design, and there was almost no influence of diet. After a repeated administration of TS-141, the plasma concentration of tipepidine reached almost a steady state 3 days after the administration, and no accumulation was observed. When comparing the plasma concentration of tipepidine after the administration of Asverin, TS-141 functioned as a sustained-release preparation and was confirmed to be appropriately exposed and well tolerated. In phase I study, no protocol deviations were observed.

**Fig. 2:**
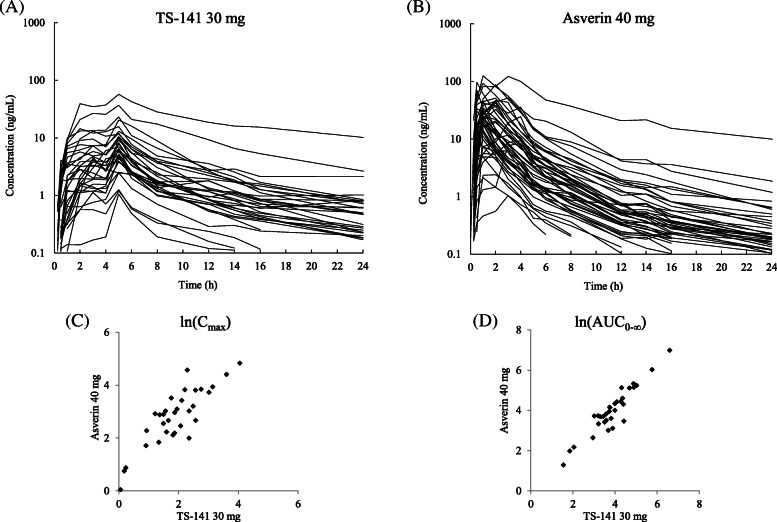
Pharmacokinetics of tipepidine in TS-141 and Asverin group according to subjects. Each single line shows the plasma concentration of tipepidine after the administration of TS-141 30 mg (**a**) or Asverin 40 mg (**b**) by each subject. The correlation of tipepidine C_max_ (ng/mL) and AUC_0-∞_ (ng⋅h/mL) in TS-141 30 mg (**c**) and Asverin 40 mg (**d**) was determined using the natural logarithm

The exposure of tipepidine in both TS-141 and Asverin groups widely varied (CV of AUC, 164% in TS-141; 155% in Asverin) among individuals; however, C_max_ and AUC were correlated between TS-141 (*n* = 32) and Asverin (*n* = 48) (Fig. 2). As a result of genetic testing of 42 subjects, individual differences on the plasma concentration of tipepidine were correlated with CYP2D6 phenotype. The distribution of polymorphisms was not different from the results of Japanese general epidemiological studies with 35 subjects with EM, 5 subjects with IM, and one subject with both UM and PM (Table 1). Table 2 shows the pharmacokinetic parameters of tipepidine in TS-141 and Asverin groups for each CYP2D6 phenotype. C_max_ and AUC_0-∞_ values when TS-141 was administered were high in the order of PM, IM, EM, and UM.

**Table 1:** CYP2D6 allele frequency at TS-141 Phase I study

CYP2D6 Phenotype	N	(%)	Allele	N	(%)
Total	42	(100.0)			
PM	1	(2.4)	*5/*5	1	(2.4)
IM	5	(11.9)	*5/*10	1	(2.4)
			*10/*10	3	(7.1)
			*10/*41	1	(2.4)
EM	35	(83.3)	*1/*1	9	(21.4)
			*1/*2	2	(4.8)
			*1/*5	2	(4.8)
			*1/*10	14	(33.3)
			*1/*10A	2	(4.8)
			*1/*41	2	(4.8)
			*2/*10	4	(9.5)
UM	1	(2.4)	*1/*2A	1	(2.4)

*A* Amplification

**Table 2:** Pharmacokinetic parameter of tipepidine in TS-141 and Asverin group

TS-141 30 mg		All Phenotypes	PM	IM	EM	UM
N		29	1	2	25	1
C_max_ (ng/mL)		9.90 ± 11.6	57.1	24.9 ± 16.8	7.17 ± 4.66	1.06
AUC_0-∞_ (ng⋅h/mL)	arithmetic mean	83.9 ± 138	728	213 ± 148	51.0 ± 35.1	4.69
	geometric mean	45.9	728	185	40.3	4.69
	CV (%)	164	–	69.5	68.8	–
MRT_0-∞_ (h)		11.8 ± 4.70	17.0	9.86 ± 0.764	11.8 ± 4.93	11.0
Asverin 40 mg		All Phenotypes	PM	IM	EM	UM
N		42	1	5	35	1
C_max_ (ng/mL)		30.7 ± 28.5	125	58.2 ± 26.2	24.9 ± 21.6	1.05
AUC_0-∞_ (ng⋅h/mL)	arithmetic mean	114 ± 177	1080	248 ± 126	70.5 ± 57.9	3.62
	geometric mean	61.9	1080	220	51.6	3.62
	CV (%)	155	–	50.8	82.2	–
MRT_0-∞_ (h)		4.67 ± 1.67	12.3	5.76 ± 0.578	4.33 ± 1.13	3.54

*PM* Poor metabolizer, *IM* Intermediate metabolizer, *EM* Extensive metabolizer, *UM* Ultrarapid metabolizer

### Phase II study

#### Study population and characteristics

**Fig. 3:**
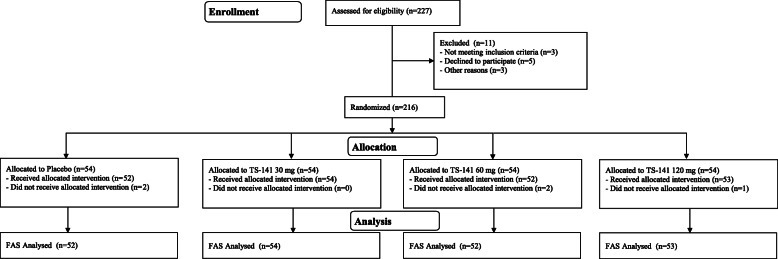
Overview of patient classification. ad: administration

Of the 227 patients who participated in the study (range for patient recruitment and follow-up: May 12, 2016 to August 16, 2017), 216 were randomized for treatment into 4 groups (Fig. 3): 211 received at least one dose of study medication (FAS), and 195 were followed up based on the study protocol (PPS). Baseline patient characteristics in all treatment groups were similar and summarized in Table 3. Mean age for each group was between 9 and 10 years with male predominant. The mean IQ was 95.3 ± 13.5. The most common type was mixed (58.3%), followed by inattentiveness (41.2). Only one patient had hyperactive-impulsive type (TS-141120 mg). About 22.7% of patients had ASD, without significant difference between the groups. About half of patients had history of previous treatment use for ADHD. The baseline ADHD RS-IV-J:I score (total score) was 33.8 ± 6.8. The percentage of CYP2D6 phenotype for each group is also shown in Table 3. Only one patient with PM was assigned to the 30 mg group. Baseline scores and changes in the primary end-point, ADHD RS-IV-J:I total score, are shown in Table 4.

**Table 3:** Demographic characteristics at baseline (FAS)

	Placebo		TS-141 30 mg		TS-141 60 mg		TS-141 120 mg		Total		*P*-value
	*n* = 52		*n* = 54		*n* = 52		*n* = 53		*n* = 211		
Gender, n (%)											
Male	44	(84.6)	47	(87.0)	46	(88.5)	42	(79.2)	179	(84.8)	p_(a)_ = 0.567
Female	8	(15.4)	7	(13.0)	6	(11.5)	11	(20.8)	32	(15.2)	
Age, years											
Mean ± SD	9.6 ± 2.1		9.8 ± 2.3		9.2 ± 2.2		9.7 ± 2.5		9.5 ± 2.3		p_(b)_ = 0.493
Median	9.0		10.0		9.0		10.0		9.0		
Min - Max	6–14		6–16		6–16		6–16		6–16		
Intelligence Quotient											
Mean ± SD	96.5 ± 14.5		96.6 ± 15.0		93.7 ± 12.6		94.4 ± 11.7		95.3 ± 13.5		p_(b)_ = 0.662
Median	95.5		94.0		92.0		93.0		93.0		
Min - Max	71–142		72–145		73–137		76–126		71–145		
ADHD Subtype, n (%)											
Combined	31	(59.6)	28	(51.9)	34	(65.4)	30	(56.6)	123	(58.3)	p_(a)_ = 0.538
Inattentive	21	(40.4)	26	(48.1)	18	(34.6)	22	(41.5)	87	(41.2)	
Hyperactive-impulsive							1	(1.9)	1	(0.5)	
Complication: ASD, n (%)											
Absence	40	(76.9)	45	(83.3)	37	(71.2)	41	(77.4)	163	(77.3)	p_(a)_ = 0.524
Presence	12	(23.1)	9	(16.7)	15	(28.8)	12	(22.6)	48	(22.7)	
Prior medication, n (%)											
Absence	20	(38.5)	27	(50.0)	27	(51.9)	31	(58.5)	105	(49.8)	p_(a)_ = 0.224
Presence	32	(61.5)	27	(50.0)	25	(48.1)	22	(41.5)	106	(50.2)	
ADHD RS-IV-J: investigator											
Mean ± SD	34.7 ± 6.4		33.4 ± 7.4		34.9 ± 7.1		32.3 ± 5.8		33.8 ± 6.8		p_(c)_ = 0.176
Median	34.0		32.0		35.0		32.0		33.0		
CYP2D6 Phenotype, n (%)											
PM			1	(1.9)					1	(0.5)	–
IM	9	(17.3)	9	(16.7)	10	(19.2)	11	(20.8)	39	(18.5)	
EM	43	(82.7)	44	(81.5)	39	(75.0)	40	(75.5)	166	(78.7)	
UM					1	(1.9)	2	(3.8)	3	(1.4)	
Unknown type/Unable to be determined					2	(3.8)			2	(0.9)	

*p*_*(a)*_ Chi-square test, *p*_*(b)*_ Kruskal-Wallis test, *p*_*(c)*_ Analysis of variance

**Table 4:** Mean changes in ADHD RS-IV-J:I over time for all treatment groups (FAS)

	Placebo	TS-141 30 mg		TS-141 60 mg		TS-141120 mg	
	(*N* = 52)	(*N* = 54)		(*N* = 52)		(*N* = 53)	
ADHD RS-IV-J:I			Difference with placebo		Difference with placebo		Difference with placebo
Start of the study (Mean ± SD)	34.7 ± 6.4	33.4 ± 7.4	–	34.9 ± 7.1	–	32.3 ± 5.8	–
Change from Baseline (Point estimate [95%CI] (*p*-value))							
Week 2	−3.8 [−5.6 - -2.1]	− 3.6 [− 5.3 - -1.9]	0.2 [− 1.8–2.2] (*p* = 0.830)	−3.1 [− 4.9 - -1.3]	0.7 [−1.3–2.7] (*p* = 0.488)	−4.2 [− 5.9 - -2.4]	−0.3 [− 2.3–1.7] (*p* = 0.735)
Week 4	−6.0 [− 8.4 - -3.6]	−4.7 [− 6.9 - -2.5]	1.3 [−1.3–3.8] (*p* = 0.321)	−3.9 [− 6.2 - -1.5]	2.1 [−0.4–4.7] (*p* = 0.104)	−6.2 [− 8.5 - -3.9]	−0.2 [− 2.8–2.3] (*p* = 0.849)
Week 8	−9.1 [− 11.9 - -6.2]	−6.9 [− 9.6 - -4.2]	2.2 [− 0.8–5.2] (*p* = 0.158)	−5.4 [− 8.1 - -2.6]	3.7 [0.6–6.7] (*p* = 0.018)	−9.0 [− 11.7 - -6.3]	0.0 [− 3.0–3.0] (*p* = 0.986)
End of the study	−8.0 [− 10.6 - -5.4]	− 6.0 [− 8.5 - -3.5]	2.0 [− 0.9–4.8] (*p* = 0.183)	−4.9 [− 7.5 - -2.3]	3.1 [0.2–6.0] (*p* = 0.037)	−8.5 [− 11.0 - -5.9]	− 0.5 [− 3.4–2.4] (*p* = 0.748)

*p* ANCOVA with baseline and CYP2D6 phenotype as covariates

#### Efficacy: primary analysis

The score decreased over time from week 2 to the end of period, week 8, in all groups. In any of the TS-141 treatment groups, no significant improvement in ADHD RS-IV-J:I total score change was observed at the end of the study as compared with the placebo group (placebo: − 8.0 [− 10.6 to − 5.4], TS-141 30 mg: − 6.0 [− 8.5 to − 3.5], TS-141 60 mg: − 4.9 [− 7.5 to − 2.3], and TS-141120 mg: − 8.5 [− 11.0 to − 5.9] at the end of the study), and no dose response was observed. Similar results were obtained for secondary end-points (data not shown).

#### Efficacy: subgroup analysis

**Fig. 4:**
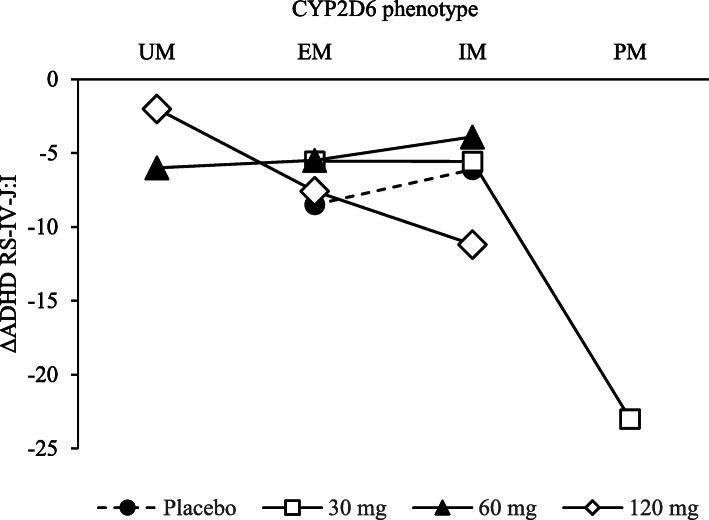
Changes from baseline to the end of treatment in ADHD RS-IV-J:I score for TS-141 (FAS). A subgroup analysis evaluated the efficacy between the dose and CYP2D6 phenotype. To compare the change for each CYP2D6 under the constant dose, lines were drawn at each dose

The ADHD RS-IV-J:I (mean ± SD) changes in the placebo and 120 mg treatment groups were − 6.1 ± 11.1 and − 11.2 ± 9.5 in CYP2D6 IM group (the slow metabolizer group), but no difference in the CYP2D6 EM group (the rapid metabolizer group) (Table 5). The total score change tended to increase from UM to PM when considering each dose curve. Moreover, each dose curve has a different slope (Fig. 4). In the CYP2D6 IM group, response rate in the placebo group and 120 mg treatment group were 33.3 and 54.6% (Table 6).

**Table 5:** Mean changes in ADHD RS-IV-J:I by CYP2D6 phenotype in the subgroup analysis (FAS)

CYP2D6 phenotype	UM	EM	IM	PM
Placebo		−8.5 ± 8.4 (43)	− 6.1 ± 11.1 (9)	
TS-141 30 mg		− 5.5 ± 7.0 (44)	− 5.6 ± 5.6 (9)	− 23.0 (1)
TS-141 60 mg	− 6.0 (1)	− 5.5 ± 6.9 (39)	− 3.9 ± 5.0 (10)	
TS-141120 mg	− 2.0 ± 4.2 (2)	− 7.6 ± 7.1 (40)	− 11.2 ± 9.5 (11)	

Mean ± SD (N)

**Table 6:** Response rate of changes in ADHD RS-IV-J:I in the subgroup analysis (FAS)

	Placebo	TS-141 30 mg	TS-141 60 mg	TS-141120 mg
All groups				
Responder /n	24/52	18/54	14/52	25/53
Response rate (%)	46.2	33.3	26.9	47.2
CYP2D6 IM				
Responder /n	3/9	4/9	2/10	6/11
Response rate (%)	33.3	44.4	20.0	54.6

### Safety

The safety of TS-141 was examined in 211 patients who received at least one dose of the study medication. The incidence of AEs was 36.5% (placebo), 51.9% (30 mg), 46.2% (60 mg), and 49.1% (120 mg), and the incidence of side effects was 3.8% (placebo), 5.6% (30 mg), 17.3% (60 mg), and 3.8% (120 mg), but no significant difference was observed between the groups (Table 7). No death occurred, and only one serious AE was reported in the placebo group. Although three patients in the TS-141 group stopped treatment because of AEs, all of them recovered and the consequence of study drug was denied. The most common side effect was somnolence, one of the side effects of Asverin, which occurred (frequency of occurrence) in 2 (3.8%), 2 (3.7%), 5 (9.6%), and 0 (0%) patients in the placebo, TS-141 30 mg, 60 mg, and 120 mg, respectively. No clinically significant changes in laboratory test, body weight, and vital signs were observed. An ECG abnormality was observed in one patient in the placebo group, but not in the TS-141 treatment groups. As a result of C-SSRS, suicidal ideation was observed in one patient in the 30 mg group after the study drug administration, but bullying from a classmate was reported and the event was clearly associated with suicidal ideation without causal relationship with the study drug and with mild symptoms. Therefore, the trial was continued. There was no clinical difference in any safety data between CYP2D6 polymorphism, including the patient with CYP2D6 PM.

**Table 7:** Summary of overall safety and adverse drug reactions during the 8 weeks treatment

	Placebo		TS-141 30 mg		TS-141 60 mg		TS-141120 mg		*P*-value
	*N* = 52	(%)	*N* = 54	(%)	*N* = 52	(%)	*N* = 53	(%)	
AE	19	(36.5)	28	(51.9)	24	(46.2)	26	(29.1)	p_(a)_ = 0.420
AE leading to death	0	(0.0)	0	(0.0)	0	(0.0)	0	(0.0)	–
Serious Adverse Event	1	(1.9)	0	(0.0)	0	(0.0)	0	(0.0)	–
AE leading to discontinuation	1	(1.9)	0	(0.0)	0	(0.0)	0	(0.0)	–
Adverse drug reactions	2	(3.8)	3	(5.6)	9	(17.3)	2	(3.8)	p_(b)_ = 0.050
Constipation					1	(1.9)			
Nausea							1	(1.9)	
Decreased appetite					1	(1.9)			
Headache			1	(1.9)					
Somnolence	2	(3.8)	2	(3.7)	5	(9.6)			
Inappropriate affect					2	(3.8)			
Insomnia					1	(1.9)			
Eczema							1	(1.9)	

*p*_*(a)*_ Chi-square test, *p*_*(b)*_ Fisher’s exact test

## Discussion

Drug repositioning is a drug development strategy predicated on the reuse of existing licensed drugs whose safety and pharmacokinetics in humans have already been confirmed. Its most significant advantages are “certainty” and “low-cost” development due to many safety existing data in clinical settings. Several drugs have already been launched through drug repositioning, such as minoxidil, thalidomide, raloxifene [27]; however, from 1980 to 2012, only 24% of repositioning drug candidates were successfully marketed and only 2% achieved success in an unrelated arena to that of the original indication [28]. In addition to lack of efficacy, many trial outcomes were falsely negative because of incorrect assumption on the dose and route of administration [1].

With this background, we also have attempted to develop a new indication for tipepidine which is already launched as an antitussive for the treatment of ADHD in children and adolescents using the drug repositioning method. In developing TS-141 as a therapeutic agent for ADHD, the lower dose of TS-141 was considered to be 30 mg with reference to two previous open studies [4–6]. These trials have reported its efficacy in pediatric patients with ADHD at the same dose as the original indication of antitussives (30 mg/day). In a recently reported Iranian placebo-controlled study, in which tipepidine is added to methylphenidate, its effect on ADHD was confirmed at the same dose as antitussives, although the study design was different from that of the TS-141 phase II study [29]. Conversely, the upper dose was set to 120 mg, the maximum dose of Asverin to reduce the clinical data package for application using the abundant safety profile of Asverin.

At first, to investigate the pharmacokinetics of TS-141, the phase I study was conducted. In phase I study, TS-141 was confirmed to show the sustained-release profile of tipepidine, and no AEs occurred in Japanese healthy volunteers. The maximum dose of TS-141 was set to 120 mg (60 mg twice a day) as a dose that does not exceed the exposure of tipepidine after the administration of Asverin 120 mg. However, the plasma concentration of tipepidine in both administrations of TS-141 and Asverin revealed a large inter-individual difference among subjects. The scatter plot of the C_max_ and AUC_0-∞_ of tipepidine in each subject showed a straight line, correlation coefficient both close to 1, suggesting that the exposure of tipepidine in each subject was strongly related with the administrations of TS-141 and Asverin. Therefore, variation in the plasma exposure was confirmed to be due to the difference in metabolic enzyme activity for each subject.

Asverin was approved in 1959, at which time the approval requirements were different and the metabolic pathway was not specified. In order to estimate the CYP molecular species involved in the metabolism of tipepidine, the inhibition rate was calculated from the residual rate after its incubation with human liver microsomes in the presence of each CYP inhibitor after the phase I study (data not shown). The metabolism of tipepidine by human liver microsomes was almost equally inhibited by potent CYP2D6 inhibitors and nonspecific CYP inhibitors; however, other CYP inhibitors did not inhibit tipepidine metabolism, estimating that CYP2D6 was mainly involved in the tipepidine metabolism.

As a result of the CYP2D6 phenotype classification from the CYP2D6 genotype in the phase I post hoc study, the ratio of each phenotype was almost the same as the survey report in Japanese studies (PM: 0.5%, IM: 18.3%, EM: 77.5%, UM: very rare) [30]. The C_max_ and AUC_0-∞_ of tipepidine in the administration of TS-141 30 mg or Asverin 40 mg showed high exposure in the order of PM, IM, EM, and UM, which were inversely correlated with metabolic enzyme activity. The reason of the variation was considered to be caused by the enzyme phenotype of CYP2D6 in individuals. The inter-individual difference (CV%) of tipepidine exposure was larger than that of atomoxetine [31] in extremely high impact and contribution of CYP2D6 metabolism. Atomoxetine is also metabolized by CYP2D6, and another metabolic pathway is suspected.

In the phase II study, changes in the ADHD RS-IV-J:I total score were not significantly different between any TS-141 treatment groups and the placebo group, and no dose response was observed in the TS-141 30, 60, 120 mg. However, the total ADHD RS-IV-J:I score decreased in all groups, confirming some impacts on ADHD symptoms. Further in the subgroup analysis, ADHD RS-IV-J:I in the low metabolizer group (CYP2D6 IMs) in placebo group, − 6.1 ± 11.1 (*n* = 9) vs. the dose of TS-141120 mg was − 11.2 ± 9.5 (*n* = 11). The effect size between the placebo and 120 mg group in the low metabolizer group (CYP2D6 IMs) was 0.49, i.e., higher than that of atomoxetine (0.44; calculated from published data [15]). Furthermore, response rate in CYP2D6 IMs TS-141120 mg group was greater than placebo group. These data suggested that TS-141 can potentially improve ADHD, and higher dose effects of TS-141 are expected for CYP2D6 EMs with ADHD patients, accounting for 80% of the Japanese population. In addition, considering the total score change in Fig. 4, each dose curve tended to be increasing from UM to PM, and the slopes were not constant. Therefore, tipepidine concentration in the plasma and ADHD RS-IV-J:I changes are correlated. Changes in tipepidine exposure under the interaction of TS-141 dose and CYP2D6 phenotype affect the ADHD RS-IV-J:I change.

In the process of conducting the phase I study, individual differences in metabolic enzyme activity were found to have an effect on exposure. Results of these studies revealed that genetic polymorphisms of drug-metabolizing enzymes that affect the pharmacokinetics, especially for old drugs with little PK information, should be examined in advance. Results of the phase II study suggested that the optimal dose of tipepidine for ADHD was not the same as that of antitussive. Therefore, the dose should be set according to the indication due to the difference in the mechanism, e.g., methotrexate 5–10 mg/day for leukemia vs. 6 mg/week for rheumatism. As described above, the dose in the phase II study was determined based on previous open studies and a phase I study; however, results of the phase II study suggest that these concepts were not sufficient for dose setting. In our case, the problem was assuming that it was effective at ≥30 mg. In the drug repositioning development, dose setting from previous pilot studies is one of the useful approaches; however, the dose determination from only open, small, or adjunctive design trials has a risk of false study.

This report showed a drug repositioning approach of TS-141 and a sustained-release tablet of tipepidine for the treatment of ADHD. This study revealed that ADHD RS-IV-J changes depend on the exposure of tipepidine in the plasma, determined according to the administration dose and CYP2D6 phenotype. For ADHD medication, higher dose than antitussives will be suspected. Considering these results, the safety and efficacy of TS-141 at high dose should be verified with a new study design protocol. Our study suggests that dose setting is important for other indications in the drug repositioning strategy, and the approved dose should be adjusted with careful consideration on the genetic polymorphism of metabolic enzymes.

The limitation of this study was the low population, low proportion of CYP2D6 phenotype, heterogeneous proportion of ADHD, fixed-dose parallel design, and 8-weeks treatment. Further studies with larger sample size, long term administration or flexible dose administration will be necessary to estimate safety and efficacy of TS-141.

## Conclusions

Changes in ADHD RS-IV-J on TS-141 may depend on the CYP2D6 phenotype, suggesting that further clinical trials should be conducted with careful consideration of the polymorphism. This drug repositioning approach was attempted at the same dose as that of the antitussive; however, the dose setting based on the indication was necessary.

## Supplementary Information

**Additional file 1.** List of the ethics committees.

## Data Availability

The data that support the findings of this study are available from Taisho Pharmaceutical co., ltd., but restrictions apply to the availability of these data, which were used under license for the current study, and so are not publicly available. Data are however available from the authors upon reasonable request and with permission of Taisho Pharmaceutical co., ltd.

## Abbreviations

ADHD: Attention-deficit/hyperactivity disorder
ADHD RS-IV-J: ADHD Rating Scale IV Japanese version
AEs: Adverse events
ANCOVA: Analysis of covariance
ASD: Autism spectrum disorder
CGI-ADHD-S: Clinical Global Impression-Severity of ADHD
C-SSRS: Columbia-suicide severity rating scale
CYP2D6: Cytochrome P450
DSM-5: Diagnostic and Statistical Manual of Mental Disorders, Fifth Edition
ECGs: Electrocardiograms
EM: Extensive metabolizer
FAS: Full analysis set
GIRK: G-protein-activated inwardly rectifying potassium
IM: Intermediate metabolizer
IQ: Intelligence quotient
LC-MS/MS: Liquid chromatography-tandem mass spectrometry
PM: Poor metabolizer
PPS: Per protocol set
QCD: Questionnaire-Children with Difficulties
QOL: Quality of life
UM: Ultrarapid metabolizer
