# Supplementary material for: Using the drug repositioning approach to develop a novel therapy, tipepidine hibenzate sustained-release tablet (TS-141), for children and adolescents with attention-deficit/hyperactivity disorder
Source: BMC Psychiatry. 2020 Nov 10;20:530. doi: 10.1186/s12888-020-02932-2 (PMC7653993; doi:10.1186/s12888-020-02932-2)
Supplement: Supplementary file 1 — Additional file 1. List of the ethics committees. [file 12888_2020_2932_MOESM1_ESM.docx]

**List of the ethics committees**

| No. | Site | Institutional review board |
| --- | --- | --- |
| 1 | Sumida Hospital | Hakata Clinic IRB |
| 2 | Hokkaido Ujiie Clinic for Psychosomatic Children | Sapporo Medical Association's IRB |
| 3 | Tokiwa Hospital | Kyoyukai RiverSide Clinic Institutional Review Board |
| 4 | Hokkaido University Hospital | Hokkaido University Hospital Institutional Review Board |
| 5 | Maeta Shounika Clinic | Oikawa Clinic IRB |
| 6 | Igarashi Children's Clinic | Oikawa Clinic IRB |
| 7 | Ken Clinic | Iwata body’s clinic Institutional Review Board |
| 8 | Hello Clinic | Oikawa Clinic IRB |
| 9 | Kohnodai Hospital | Kohnodai hospital, National Center for Global Health and Medicine |
| 10 | National Center of Neurology and Psychiatry | National Center of Neurology and Psychiatry Institutional Review Board |
| 11 | Hattatsu Shinryo Clinic | Meiwa Hospital Institutional Review Board |
| 12 | Tokyo Nishi Tokushukai Hospital | The Tokushukai Group Ethics Committee |
| 13 | Shimada Ryoiku Center | Japan Conference of Clinical Research |
| 14 | Ohwamental Clinic | The Institutional Review Board at Yoyogi Mental Clinic |
| 15 | Oyamadai Suku-Suku Clinic | Mizuo Clinic Institutional Review Board |
| 16 | Tokyo Metropolitan Children's Medical Center | TOKYO METROPOLITAN CHILDREN'S MEDICAL CENTER Institutional Review Board |
| 17 | Aiiku Clinic | Suzuki Internal & Circulatory Medical Clinic Institutional Review Board |
| 18 | Micri Kids Clinic | Mizuo Clinic Institutional Review Board |
| 19 | Tokyo Metropolitan Health and Medical Treatment Corporation Tama-Hokubu Medical Center | Tama-Hokubu Medical Center Institutional Review Board |
| 20 | The University of Tokyo Hospital | Review Board of The University of Tokyo Hospital |
| 21 | Yokohama Onoecho Clinic | The Institutional Review Board at Yoyogi Mental Clinic |
| 22 | Inoko Mental Clinic | The Institutional Review Board at Yoyogi Mental Clinic |
| 23 | Kanagawa Children's Medical Center | Kanagawa children's medical centre |
| 24 | St. Marianna Association Toyoko Keiai Hospital | Shinagawa East one Medical Clinic Institution Review Board |
| 25 | Tokai University Hospital | Tokai Universtity Hospital group IRB |
| 26 | Niigata mental and developmental clinic | Nagaoka Nishi Hospital Institutional Review Board |
| 27 | Toyama Rehabilitation Hospital/Child Support Care Center | Toyama Medical Association Institutional Review Board |
| 28 | University of Fukui Hospital | University of Fukui Hospital Institutional Review Board |
| 29 | Hiratani Child Development Clinic | University of Fukui Hospital Institutional Review Board |
| 30 | Nagoya university Hospital | Nagoya University Hospital Institutional review board |
| 31 | Seikeikai Hospital | Seikeikai Hospital Institutional Review Board |
| 32 | Suzuki Clinic | Japan Conference of Clinical Research |
| 33 | Kusube Mental Clinic | Meiwa Hospital Institutional Review Board |
| 34 | Yasuhara Children`s Clinic | Chibune General Hospital Institutional Review Board |
| 35 | Osaka City General Hospital | Osaka City General Hospital, Institutional Review Board |
| 36 | Mikuni-hill Mental Clinic | Ekihigashi Hifuka Clinic IRB |
| 37 | Takahashi Psychiatric Clinic | Tokyo Institute of Allergy and Respiratory Disease Institutional Review Board |
| 38 | Hibiki Mental Clinic | Kondo Memorial Medical Foundation　Tomisaka Clinic Institutional Review Board |
| 39 | Kyo Mental Clinic | Dr.Mano Medical Clinic IRB |
| 40 | Nara Medical University Hospital | IRB in Nara Medical Universtity |
| 41 | NHO Minami Wakayama Medical Center | National Hospital Organization Minami Wakayama Medical Center Institutional Review Board |
| 42 | Ohno Hagukumi Clinic | Dr.Mano Medical Clinic IRB |
| 43 | Manaboshi Clinic | Oikawa Clinic IRB |
| 44 | Hiroshima Nishi Medical Center | Hiroshima-Nishi Medical center, Institutional Review Board |
| 45 | Japanese Red Cross Tokushima Hinomine Rehabilitation Center for people with Disabilities | Oikawa Clinic IRB |
| 46 | Kurume University Hospital | Oikawa Clinic IRB |
| 47 | Sansuikai Kashii Mental Hospital | Hakataeki-higashi Clinic, Institutional Review Board |
| 48 | Fukuoka University Chikushi Hospital | FUKUOKA UNIVERSITY CHIKISHI HOSPITAL IRB |
| 49 | Kyushu University Hospital | KYUSHU UNIVERSITY HOSPITAL IRB |
| 50 | Rainbow & Sea Hospital | Ekihigashi Hifuka Clinic IRB |
| 51 | NHO Hizen Psychiatric Center | Hizen Psychiatric Center IRB |
| 52 | Koyodai Hospital | Dr.Mano Medical Clinic IRB |
| 53 | NHO Ryukyu Hospital | National Hospital Organization Ryukyu Hospital Institutional Review Board |
| 54 | Neurodevelopment clinic prop | Okinawa Medical Association IRB |
